# Insights from Classifying Visual Concepts with Multiple Kernel Learning

**DOI:** 10.1371/journal.pone.0038897

**Published:** 2012-08-24

**Authors:** Alexander Binder, Shinichi Nakajima, Marius Kloft, Christina Müller, Wojciech Samek, Ulf Brefeld, Klaus-Robert Müller, Motoaki Kawanabe

**Affiliations:** 1 Machine Learning Group, Berlin Institute of Technology, Berlin, Germany; 2 Fraunhofer Institute FIRST, Berlin, Germany; 3 Optical Research Laboratory, Nikon Corporation, Tokyo, Japan; 4 Knowledge Discovery and Machine Learning Group, University of Bonn, Bonn, Germany; 5 Zalando GmbH, Berlin, Germany; 6 Bernstein Focus: Neurotechnology Berlin, Berlin, Germany; 7 Department of Brain and Cognitive Engineering, Korea University, Anam-dong, Seongbuk-gu, Seoul, Korea; 8 ATR Research, Kyoto, Japan; University of East Piedmont, Italy

## Abstract

Combining information from various image features has become a standard technique in concept recognition tasks. However, the optimal way of fusing the resulting kernel functions is usually unknown in practical applications. Multiple kernel learning (MKL) techniques allow to determine an optimal linear combination of such similarity matrices. Classical approaches to MKL promote sparse mixtures. Unfortunately, 1-norm regularized MKL variants are often observed to be outperformed by an unweighted sum kernel. The main contributions of this paper are the following: we apply a recently developed non-sparse MKL variant to state-of-the-art concept recognition tasks from the application domain of computer vision. We provide insights on benefits and limits of non-sparse MKL and compare it against its direct competitors, the sum-kernel SVM and sparse MKL. We report empirical results for the PASCAL VOC 2009 Classification and ImageCLEF2010 Photo Annotation challenge data sets. Data sets (kernel matrices) as well as further information are available at http://doc.ml.tu-berlin.de/image_mkl/(Accessed 2012 Jun 25).

## Introduction

A common strategy in visual object recognition tasks is to combine different image features to capture relevant traits of an image. Prominent features are, for instance, built from color, texture, and shape information and used to accurately locate and classify the objects of interest. The importance of such image features changes across the tasks. For example, color information increases the detection rates of stop signs in images substantially but it is almost useless for finding cars. This is because, in most countries, stop signs are red, while cars can have any color. Additional less informative features may not only slow down the computation time, but even can harm the predictive performance by adding noise to the resulting classifier. Therefore it is necessary to exclude insufficiently informative features in order to achieve the predictive performance of state-of-the-art (SOTA) object recognition systems (by “SOTA systems'” we here refer to top-ranked submissions in established annual benchmark challenges such as Pascal VOC (http://pascallin.ecs.soton.ac.uk/challenges/VOC/, Accessed 2012 Jun 25) [1], ImageCLEF (http://www.imageclef.org/, Accessed 2012 Jun 25) as well as TRECVID (http://trecvid.nist.gov/, Accessed 2012 Jun 25) [2] for video data). This raises the question how a combination of features can be learned from the available data.

In this paper, we approach visual object classification from a machine learning perspective. In the past decade, support vector machines (SVM) [3]–[5] have been successfully applied to many practical problems in various application fields including computer vision [6]. Support vector machines exploit similarities of the data, arising from some (possibly nonlinear) measure. The matrix of pairwise similarities, also known as *kernel matrix*, allows to abstract the data from the learning algorithm [7], [8].

However, the problem remains, given a task at hand, to find an appropriate similarity measure and to plug the resulting kernel into an appropriate learning algorithm. But what if this similarity measure is difficult to find? We note that [9] and [10] were the first to exploit prior and domain knowledge for the kernel construction.

In object recognition, translating information from various features into several kernels has now become a standard technique. Consequently, the choice of finding the right kernel changes to finding an appropriate way of fusing the kernel information; however, finding the right combination for a particular application is so far often a matter of a judicious choice (or trial and error).

In the absence of principled approaches, practitioners frequently resort to heuristics such as uniform mixtures of normalized kernels [11], [12] that have proven to work well. Nevertheless, this may lead to sub-optimal kernel mixtures.

An alternative approach is multiple kernel learning (MKL), which has been applied to object classification tasks involving various image features [13], [14]. Multiple kernel learning [15]–[18] generalizes the support-vector-machine framework and aims at *simultaneously* learning the optimal kernel mixture *and* the model parameters of the SVM. To obtain a well-defined optimization problem, many MKL approaches promote sparse mixtures by incorporating a 1-norm constraint on the mixing coefficients. Compared to heuristic approaches, MKL has the appealing property of automatically selecting kernels in a mathematical sound way and converges quickly as it can be wrapped around a regular support vector machine [17]. However, some evidence shows that sparse kernel mixtures are often outperformed by an unweighted-sum kernel [19]. As a remedy, [20], [21] propose 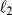-norm regularized MKL variants, which promote non-sparse kernel mixtures and subsequently have been extended to 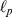-norms [22], [23].

Multiple Kernel approaches have been applied to various computer vision problems outside our scope such multi-class problems [24], which require in distinction to the general multi-label case mutually exclusive labels and object detection [25], [26] in the sense of finding object regions in an image. The latter reaches its limits when image concepts cannot anymore be represented by an object region such as the *Outdoor*,*Overall Quality* or *Boring* concepts in the ImageCLEF2010 dataset that we will use. Please note that we make a distinction between the general case of multi-label classification and the more special case of multi-class classification with mutually exclusive classes.

In this contribution, we study the benefits of sparse and non-sparse MKL in object recognition tasks. We report on empirical results on image data sets from the PASCAL visual object classes (VOC) 2009 [27] and ImageCLEF2010 PhotoAnnotation [28] challenges, showing that non-sparse MKL significantly outperforms the uniform mixture and 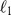-norm MKL. Furthermore, we discuss the reasons for performance gains and performance limitations obtained by MKL based on additional experiments using real world and synthetic data.

The family of MKL algorithms is not restricted to SVM-based ones. Another competitor, for example, is Multiple Kernel Learning based on Kernel Discriminant Analysis (KDA) [29], [30]. The difference between MKL-SVM and MKL-KDA lies in the underlying single kernel optimization criterion while the regularization over kernel weights is the same.

Further competitors include, for example, [31], who use logistic regression as base criterion; their approach results in a number of optimization parameters equal to the number of samples times the number of input features. Since the approach in [31] a priori uses much more optimization variables, it poses a more challenging and potentially more time consuming optimization problem, which limits the number of applicable features.

Further alternatives use more general combinations of kernels such as products with kernel widths as weighting parameters [32], [33]. As [33] point out, the corresponding optimization problems are no longer convex. Consequently, they may find suboptimal solutions and it is more difficult to assess using how much gain can be achieved by learning the kernel weights.

This paper is organized as follows. We first briefly review the machine learning techniques used in his paper. Then we present our experimental results on the VOC2009 and ImageCLEF2010 datasets, and, finally, we discuss promoting and limiting factors of MKL and the sum-kernel SVM in various learning scenarios.

## Methods

This section briefly introduces multiple kernel learning (MKL). For an extensive treatment see the survey of [34].

Given a finite number 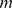 of different kernels each of which implies the existence of a feature mapping 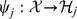 onto a Hilbert space
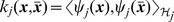
the goal of multiple kernel learning is to learn SVM parameters 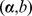 and kernel weights 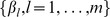 for a linear combination of these 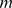 kernels 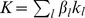 simultaneously.

This can be cast as the following optimization problem which reduces to support vector machines [4], [8] in the special case of on kernel 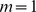


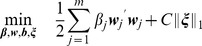
(1)

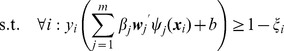


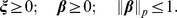
The usage of kernel mixtures 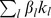 is permitted through its partially dualized form:
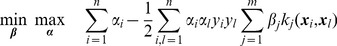
(2)

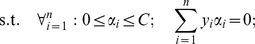


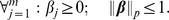
For details on the solution of this optimization problem and its kernelization we refer to [23]. This optimization problem has two parameters: the regularization constant 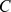 and a parameter 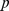 on the constraint for the kernel weights 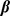. The regularization constant is known from support vector machines; it balances the margin term 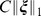 from equation (1) over the regularization term 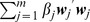. A high value of the regularization constant 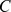 puts more emphasis on achieving high classification margins 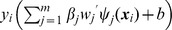 on the training data while a low value emphasizes the regularization term as a measure against overfitting on training data.

While prior work on MKL imposes a 1-norm constraint on the mixing coefficients to enforce sparse solutions lying on a standard simplex [16], [17], [35], [36], we employ a generalized 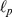-norm constraint 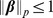 for 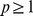 as used in [22], [23]. The implications of this modification in the context of image concept classification will be discussed throughout this paper.

## Results

In this section, we evaluate 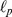-norm MKL in real-world image categorization tasks, experimenting on the VOC2009 and ImageCLEF2010 data sets. We also provide insights on *when* and *why* 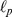-norm MKL can help performance in image classification applications. The evaluation measure for both datasets is the average precision (AP) over all recall values based on the precision-recall (PR) curves.

### Data Sets

We experiment on the following data sets:

1. **PASCAL2 VOC Challenge 2009:** We use the official data set of the *PASCAL2 Visual Object Classes Challenge 2009* (VOC2009) [27], which consists of 13979 images. The use the official split into 3473 training, 3581 validation, and 6925 test examples as provided by the challenge organizers. The organizers also provided annotation of the 20 objects categories; note that an image can have multiple object annotations. The task is to solve 20 binary classification problems, i.e. predicting whether at least one object from a class 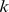 is visible in the test image. Although the test labels are undisclosed, the more recent VOC datasets permit to evaluate AP scores on the test set via the challenge website (the number of allowed submissions per week being limited).
2. **ImageCLEF 2010 PhotoAnnotation:** The ImageCLEF2010 PhotoAnnotation data set [28] consists of 8000 labeled training images taken from flickr and a test set with recently disclosed labels. The images are annotated by 93 concept classes having highly variable concepts—they contain both well defined objects such as *lake, river, plants, trees, flowers,* as well as many rather ambiguously defined concepts such as *winter, boring, architecture, macro, artificial, motion blur,* —however, those concepts might not always be connected to objects present in an image or captured by a bounding box. This makes it highly challenging for any recognition system. Unfortunately, there is currently no official way to obtain test set performance scores from the challenge organizers. Therefore, for this data set, we report on training set cross-validation performances only. As for VOC2009 we decompose the problem into 93 binary classification problems. Again, many concept classes are challenging to rank or classify by an object detection approach due to their inherent non-object nature. As for the previous dataset each image can be labeled with multiple concepts.

### Image Features and Base Kernels

In all of our experiments we deploy 32 kernels capturing various aspects of the images. Our choice of features is inspired by the VOC 2007 winner [37] and our own experiences from our submissions to the VOC2009 and ImageCLEF2009 challenges. It is known from the top-ranked submissions in recent Pascal VOC Classification and ImageCLEF PhotoAnnotation Challenges that Bag-of-Words features are necessary for state-of-the-art performance results when the focus lies on visual concept classification and ranking. At the same time adding simpler features together with multiple kernel learning may improve the ranking performance for some visual concepts as well as the average performance measured over all visual concepts (shown in [38]). For the ImageCLEF2010 dataset the test data annotations have been disclosed and we checked that adding the simpler features listed below improves, indeed, the average-kernel performance compared to relying on BoW-S features (see next section) alone. Our choice of features was furthermore guided by the intention to have several different feature types that empirically have been proven to be useful and to use gradient and color information. Furthermore the features should have reasonable computation times without the need for excessive tuning of many parameters and they should be able to capture objects and visual concept cues of varying sizes and positions. For this reason, we used bag of word features and global histograms based on color and gradient information.

The features used in the following are derived from histograms that a priori contain *no spatial information*. We therefore enrich the respective representations by using regular spatial tilings 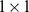, 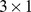, 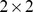, 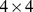, 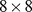, which correspond to single levels of the pyramidal approach in [11]. Furthermore, we apply a 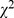 kernel on top of the enriched histogram features, which is an established kernel for capturing histogram features [12]. The bandwidth of the 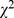 kernel is thereby heuristically chosen as the mean 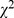 distance over all pairs of training examples, as done, for example, in [39].

**Histogram over a bag of visual words over SIFT features (BoW-S).** Histograms over a bag of visual words over SIFT features are known to yield excellent performance for visual concept recognition both when used as single features alone as well as in combination with other features. This can be observed by checking the top-ranked submissions in the recent ImageCLEF PhotoAnnotation and Pascal VOC Classification challenges and noting their general usage in publications on visual concept ranking. It has also recently been successfully deployed to object detection [40] on a large data set of images within the Imagenet Large Scale Visual Recognition Challenge.

The BoW features [41] were constructed with parameters that were established in past image annotation challenges so as to yield good results. At first, the SIFT features [42] were calculated on a regular grid with six pixel pitch for each image. We computed the SIFT features over the following color combinations, which are inspired by [43]: red-green-blue (RGB), normalized RGB, gray-opponentColor1–opponentColor2, and gray-normalized OpponentColor1–OpponentColor2; in addition, we also use a simple gray channel. For visual words we used a code book of size 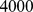 obtained by 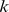-means clustering (with a random initialization of centers and using 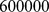 local features taken randomly from the training set). Finally, all SIFT features were assigned to the visual words (so-called *prototypes*) by adding a constant to the nearest visual word and then summarized into histograms within entire images or sub-regions. The BoW feature was normalized to an 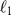-norm of one. Note that five color channel sets times three spatial tilings (see below) 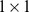, 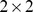 and 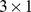 yield 15 features in total.

**Histogram over a bag of visual words over color intensity histograms (BoW-C).** This feature has been computed in a similar manner as the BoW-S feature. However, for the local feature, we employed low-dimensional color histograms instead of SIFT features, which combines the established BoW computation principle of aggregating local features into a global feature with color intensity information – this was our motivation for employing them. The color histograms were calculated on a regular grid with nine pixel pitch for each image over a descriptor support of radius 12 and histogram dimension 15 per color channel (SIFT: 128). We computed the color histograms over the following color combinations, again inspired by [43]: red-green-blue (RGB), gray-opponentColor1-opponentColor2, gray only and, finally, the hue weighted by the grey value in the pixels. For the latter the weighting implies that the hue receives a higher weight in bright pixels as a countermeasure against the known difficulties to estimate hue in dark regions of an image.

For visual words we used a code book of size 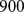 obtained by 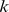-means clustering. The lower dimensionality in local features and visual words yielded a much faster computation compared to the BoW-S feature. Otherwise we used the same settings as for BoW-S. Four color channel sets times two spatial tilings 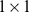 and 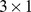 resulted in 8 BoW-C features in total.

**Histogram of oriented gradients (HoG).** The histogram of oriented gradients has proven to be useful [44] on the seminal Caltech101 Dataset [45]. It serves as an alternative and much faster way to incorporate gradient information compared to the BoW-S features. The HoG feature is based on discretizing the orientation of the gradient vector at each pixel into bins and then summarizing the discretized orientations into histograms within image regions [46]. Canny detectors [47] are used to discard contributions from pixels, around which the image is almost uniform. We computed HoG features over the following color channel combinations: red-green-blue (RGB), gray-opponentColor1-opponentColor2 and gray only, every time using 24 histogram bins for gradient orientations for each color channel and spatial tilings 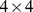 and 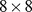.

In the experiments we deploy four kernels: a product kernel created from the two kernels with different spatial tilings with colors red-green-blue, a product kernel created from the two kernels having color combination gray-opponentColor1-opponentColor2, and the two kernels using the gray channel alone (differing in their spatial tiling). Note that building a product kernel out of 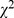 kernels boils down to concatenating feature blocks (but using a separate kernel width for each feature block).

This choice allows to employ gradient information for a specific color channel set – independent of spatial resolution – via the first two kernels and for a specific spatial resolution (independent of color channels) via the last two kernels. This is a principled way to yield diverse features: one subset varies over color channel sets and the other over spatial tilings. In total we have four HoG features.

**Histogram of pixel color intensities (HoC).** The histogram of color intensities is known to be able to improve ranking performance of BoW-S features as shown in [38], which motivated us to use it here. The HoC features were constructed by discretizing pixel-wise color values and computing their bin histograms within image regions. We computed HoC features over the following color channel combinations: red-green-blue (RGB), gray-opponentColor1-opponentColor2 and gray only, every time using 15 histogram bins for color intensities for each color channel and spatial tilings 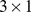, 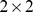 and 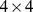.

In the experiments we deploy five kernels: a product kernel created from the three kernels with different spatial tilings with colors red-green-blue, a product kernel created from the three kernels with color combination gray-opponentColor1-opponentColor2, and the three kernels using the gray channel alone (differing in their spatial tiling). Again, please note the relation between feature concatenation and taking the product of 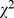-kernels. The last three kernels are HoC features from the gray channel and the two spatial tilings. This choice allows to employ color information for a specific color channel set independent of spatial resolution via the first two kernels and for a specific spatial resolution independent of color channels via the last two kernels. In total we have five HoC features.

For the HoG and HoC feature we used higher spatial tilings because these two features are much faster to compute compared to BoW features, thus allowing to increase their dimensionality by the spatial tilings, and due to our empirical experience that choices of finer spatial tilings beyond 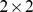 tend to yield a higher improvement for such simpler features as compared to BoW-based features.

**Summary.** We can summarize the employed kernels by the following types of basic features:

- Histogram over a bag of visual words over SIFT features (BoW-S), 15 kernels
- Histogram over a bag of visual words over color intensity histograms (BoW-C), 8 kernels
- Histogram of oriented gradients (HoG), 4 kernels
- Histogram of pixel color intensities (HoC), 5 kernels.

We used a higher fraction of bag-of-word-based features as we knew from our challenge submissions that they have a better performance than global histogram features. The intention was, however, to use a variety of different feature types that have been proven to be effective on the above datasets in the past—but at the same time obeying memory limitations of maximally ca. 25 GB per job as required by computer facilities used in our experiments (we used a cluster of 23 nodes having in total 256 AMD64 CPUs and with memory limitations ranging in 32–96 GB RAM per node).

In practice, the normalization of kernels is as important for MKL as the normalization of features is for training regularized linear or single-kernel models. Optimal feature/kernel weights are requested to be small by the 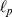-norm constraint in the optimization problem given by equation (1), implying a bias to towards excessively up-scaled kernels. In general, there are several ways of normalizing kernel functions. We apply the following normalization method, proposed in [48], [49] and entitled *multiplicative normalization* in [23];
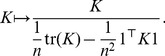
(3)The denominator is an estimator of the variance in the embedding Hilbert space computed over the given dataset 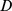 by replacing the expectation operator 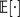 by the discrete average over the data points 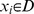.
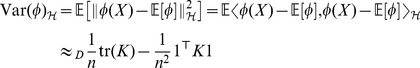
(4)Thus dividing the kernel matrix 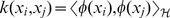 by this term is equivalent to dividing each embedded feature 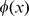 by its standard deviation over the data. This normalization corresponds to rescaling the data samples to unit variance in the Hilbert space used for SVM and MKL classification.

### Experimental Setup

We treat the multi-label data set as binary classification problems, that is, for each object category we trained a one-vs.-rest classifier. Multiple labels per image render multi-class methods inapplicable as these require mutually exclusive labels for the images. The classifiers used here were trained using the open sourced Shogun toolbox http://www.shogun-toolbox.org (Accessed 2012 Jun 25) [50]. In order to shed light on the nature of the presented techniques from a statistical viewpoint, we first pooled all labeled data and then created 20 random cross-validation splits for VOC2009 and 12 splits for the larger dataset ImageCLEF2010.

For each of the 12 or 20 splits, the training images were used for learning the classifiers, while the SVM/MKL regularization parameter 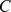 and the norm parameter 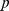 were chosen based on the maximal AP score on the validation images. Thereby, the regularization constant 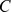 is optimized by class-wise grid search over 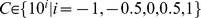. Preliminary runs indicated that this way the optimal solutions are attained inside the grid. Note that for 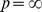 the 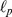-norm MKL boils down to a simple SVM using a uniform kernel combination (subsequently called sum-kernel SVM). In our experiments, we used the average kernel SVM instead of the sum-kernel one. This is no limitation in this as both lead to identical result for an appropriate choice of the SVM regularization parameter.

For a rigorous evaluation, we would have to construct a separate codebook for each cross validation split. However, creating codebooks and assigning features to visual words is a time-consuming process. Therefore, in our experiments we resort to the common practice of using a single codebook created from all training images contained in the official split. Although this could result in a slight overestimation of the AP scores, this affects all methods equally and does not favor any classification method more than another—our focus lies on a *relative* comparison of the different classification methods; therefore there is no loss in exploiting this computational shortcut.

### Numerical Evaluation

In this section we report on the empirical results achieved by 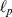-norm MKL in our visual object recognition experiments.

**VOC 2009** Table 1 shows the AP scores attained on the official test split of the VOC2009 data set (scores obtained by evaluation via the challenge website). The class-wise optimal regularization constant has been selected by cross-validation-based model selection on the training data set. We can observe that non-sparse MKL outperforms the baselines 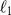-MKL and the sum-kernel SVM in this sound evaluation setup. We also report on the cross-validation performance achieved on the training data set (Table 2). Comparing the two results, one can observe a small overestimation for the cross-validation approach (for the reasons argued in Section *Experimental Setup*) —however, the amount by which this happens is equal for all methods; in particular, the ranking of the compared methods (SVM versus 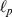-norm MKL for various values of 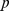) is preserved for the average over all classes and most of the classes (exceptions are the bottle and bird class); this shows the reliability of the cross-validation-based evaluation method in practice. Note that the observed variance in the AP measure across concepts can be explained in part by the variations in the label distributions across concepts and cross-validation splits. Unlike for the AUC measure [51] which is also commonly used for the evaluation of rankings of classifier predictions, the average score of the AP measure under randomly ranked images depends on the ratio of positive and negative labeled samples.

**Table 1:** AP scores on VOC2009 test data with fixed 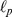-norm.

	average	aeroplane	bicycle	bird	boat	bottle	bus
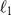	54.58	**81.13**	54.52	56.14	62.44	**28.10**	**68.92**
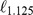	56.43	81.01	56.36	58.49	62.84	25.75	68.22
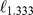	**56.70**	80.77	**56.79**	**58.88**	63.11	25.26	67.80
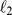	56.34	80.41	56.34	58.72	**63.13**	24.55	67.70
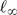	55.85	79.80	55.68	58.32	62.76	24.23	67.79

AP scores were obtained on request from the challenge organizers due to undisclosed annotations. Regularization constants were selected via AP scores computed via cross-validation on the training set. Best methods are marked boldface.

**Table 2:** AP scores obtained on the VOC2009 training data set with fixed 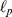-norm.

Norm	Average	Aeroplane	Bicycle	Bird	Boat	Bottle
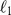	54.94±12.3	**84.84**±5.86	55.35±10.5	59.38±10.1	66.83±12.4	25.91±10.2
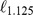	57.07±12.7	**84.82**±5.91	**57.25**±10.6	62.4±9.13	**67.89**±12.8	**27.88**±9.91
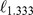	57.2±12.8	**84.51**±6.27	**57.41**±10.8	**62.75**±9.07	**67.99**±13	**27.44**±9.77
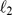	56.53±12.8	84.12±5.92	56.89±10.9	**62.53**±8.9	67.69±13	26.68±9.94
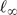	56.08±12.7	83.67±5.99	56.09±10.9	61.91±8.81	67.52±12.9	26.5±9.5

AP scores were computed by cross-validation on the training set. Bold faces show the best method and all other ones that are not statistical-significantly worse by a Wilcoxon's signed rank test with a p-value of 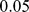.

A reason why the bottle class shows such a strong deviation towards sparse methods could be the varying but often small fraction of image area covered by bottles leading to overfitting when using spatial tilings.

We can also remark that 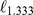-norm achieves the best result of all compared methods on the VOC dataset, slightly followed by 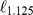-norm MKL. To evaluate the statistical significance of our findings, we perform a Wilcoxon signed-rank test for the cross-validation-based results (see Table 2; significant results are marked in boldface). We find that in 15 out of the 20 classes the optimal result is achieved by truly non-sparse 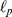-norm MKL (which means 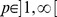), thus outperforming the baseline significantly.

**ImageCLEF** Table 3 shows the AP scores averaged over all classes achieved on the ImageCLEF2010 data set. We observe that the best result is achieved by the non-sparse 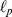-norm MKL algorithms with norm parameters 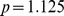 and 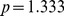. The detailed results for all 93 classes are shown in Table S1.We can see from the detailed results that in 37 out of the 93 classes the optimal result attained by non-sparse 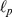-norm MKL was significantly better than the sum kernel according to a Wilcoxon signed-rank test.

**Table 3:** Average AP scores obtained on the ImageCLEF2010 test data set with 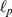-norm fixed for all classes.

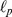 -Norm	1	1.125	1.333	2	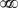
	34.61	37.01	36.97	36.62	36.45

Regularization constants were selected by AP scores computed via 12-fold cross-validation on the training set.

We also show the results for optimizing the norm parameter 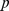 *class-wise* on the training set and measuring the performance on the test set (see Table 4 for the VOC dataset and Table 5 for the ImageCLEF dataset). We can see from Table 5 that optimizing the 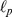-norm class-wise is beneficial: selecting the best 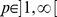 class-wise, the result is increased to an AP of 37.02—this is almost 0.6 AP better than the result for the vanilla sum-kernel SVM. Including the 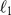-norm MKL in the candidate set results in no gains. Similarly, including the sum-kernel SVM to the set of models, the AP score does not increase compared to using 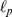-Norms in 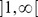 alone. A qualitatively similar result can be seen from Table 4 for the VOC 2009 dataset where we observe a gain of 0.9 AP compared to the sum-kernel SVM. We conclude that optimizing the norm parameter 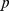 class-wise improves performance compared to the sum kernel SVM and, more importantly, model selection for the class-wise optimal 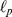-norm on the training set is stable in the sense that the choices make sense by their AP scores on the test set; additionally, one can rely on 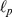-norm MKL alone without the need to additionally include the sum-kernel-SVM to the set of models. Tables 2 and 1 show that the gain in performance for MKL varies considerably on the actual concept class. The same also holds for the ImageCLEF2010 dataset.

**Table 4:** Average AP scores on the VOC2009 test data with 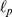-norm class-wise optimized on training data.

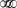	{1, 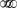 }	{1.125, 1.333, 2} 	{1.125, 1.333, 2 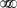 }	{1, 1.125, 1.333, 2} 	all norms from the left
55.85	55.94	56.75	56.76	56.75	56.76

AP scores on test data were obtained on request from the challenge organizers due to undisclosed annotations. The class-wise selection of 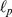-norm and regularization constant relied on AP scores obtained via cross-validation on the training set.

**Table 5:** Average AP scores on the ImageCLEF2010 test data with 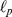-norm class-wise optimized.

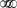	{1, 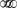 }	{1.125, 1.333, 2 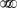 }	{1, 1.125, 1.333, 2} 	all norms from the left
36.45	37.02	37.00	36.94	36.95

The class-wise selection of 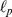-norm and regularization constant relied on AP scores obtained via cross-validation on the training set.

### Analysis and Interpretation

We now analyze the kernel set in an explorative manner; to this end, our methodological tools are the following

1. Pairwise kernel alignment scores (KKA)
2. Kernel-target alignment scores (KTA).

Both are based on measuring angles between kernel matrices embedded in a vector space and are explained briefly in the following section *Kernel Alignment*. The KKA score measures a similarity between two kernels computed from image features. The KTA score measures a similarity between one of our computed feature kernels and an optimally discriminative kernel derived from the visual concept labels.

**Kernel Alignment.** The kernel alignment introduced by [52] measures the similarity of two matrices as a cosine angle in a Hilbert space defined by the Frobenius product of matrices
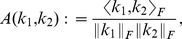
(5)It was argued in [53] that centering [54] is required in order to correctly reflect the test errors from SVMs via kernel alignment. Centering in the corresponding feature spaces is the replacement of 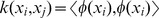 by
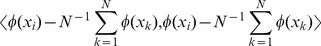
(6)Note that support vector machines using a bias term are invariant against centering, which can be shown using the condition 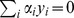 from the optimization problem given by equation (2). To see the influence of centering on kernel alignment consider that the normalized kernel alignment with an added bias 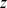 and non-negative kernels 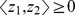 will be dominated by the bias 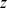 when 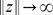:
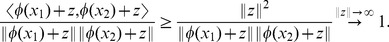
(7)Centering can be achieved by taking the product 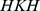, with
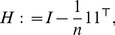
(8)

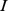
 is the identity matrix of size 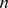 and 1 is the column vector with all ones. For kernel target alignment we will employ an optimally discriminative kernel computed from the labels for a given visual concept. The centered kernel which achieves a perfect separation of two classes can be derived from the labels and is proportional to 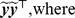


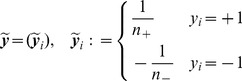
(9)and 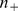 and 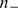 are the sizes of the positive and negative classes, respectively.

**Figure 1:**
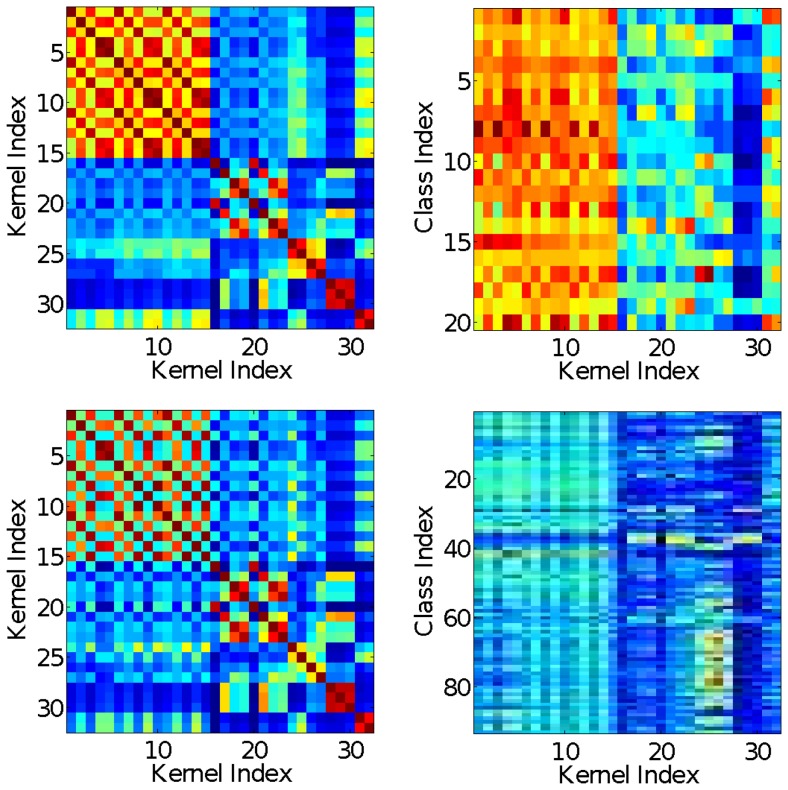
Similarity of the kernels for the VOC2009 (Top) and ImageCLEF2010 (Bottom) data sets in terms of pairwise kernel alignments (Left) and kernel target alignments (Right), respectively. In both data sets, five groups can be identified: ‘BoW-S’ (Kernels 1–15), ‘BoW-C’ (Kernels 16–23), ‘products of HoG and HoC kernels’ (Kernels 24–27), ‘HoC single’ (Kernels 28–30), and ‘HoG single’ (Kernels 31–32).

**Analysis of the Chosen Kernel Set.** To start with, we computed the pairwise kernel alignment scores of the 32 base kernels: they are shown in Figure 1. We recall that the kernels can be classified into the following groups: Kernels 1–15 and 16–23 employ BoW-S and BoW-C features, respectively; Kernels 24 to 27 are product kernels associated with the HoG and HoC features; Kernels 28–30 deploy HoC, and, finally, Kernels 31–32 are based on HoG features over the gray channel. We see from the block-diagonal structure that features that are of the same type (but are generated for different parameter values, color channels, or spatial tilings) are strongly correlated. Furthermore the BoW-S kernels (Kernels 1–15) are weakly correlated with the BoW-C kernels (Kernels 16–23). Both, the BoW-S and HoG kernels (Kernels 24–25,31–32) use gradients and therefore are moderately correlated; the same holds for the BoW-C and HoC kernel groups (Kernels 26–30). This corresponds to our original intention to have a broad range of feature types which are, however, useful for the task at hand. The principle usefulness of our feature set can be seen a posteriori from the fact that 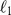-MKL achieves the worst performance of all methods included in the comparison while the sum-kernel SVM performs moderately well. Clearly, a higher fraction of noise kernels would further harm the sum-kernel SVM and favor the sparse MKL instead.

Based on the observation that the BoW-S kernel subset shows high KTA scores, we also evaluated the performance restricted to the 15 BoW-S kernels only. Unsurprisingly, this setup favors the sum-kernel SVM, which achieves higher results on VOC2009 for most classes; compared to 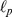-norm MKL using all 32 classes, the sum-kernel SVM restricted to 15 classes achieves slightly better AP scores for 11 classes, but also slightly worse for 9 classes. Furthermore, the sum kernel SVM, 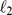-MKL, and 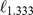-MKL were on par with differences fairly below 0.01 AP. This is again not surprising as the kernels from the BoW-S kernel set are strongly correlated with each other for the VOC data which can be seen in the top left image in Figure 1. For the ImageCLEF data we observed a quite different picture: the sum-kernel SVM restricted to the 15 BoW-S kernels performed significantly worse, when, again, being compared to non-sparse 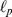-norm MKL using all 32 kernels. To achieve top state-of-the-art performance, one could optimize the scores for both datasets by considering the class-wise maxima over learning methods *and* kernel sets. However, since the intention here is not to win a challenge but a relative comparison of models, giving insights in the nature of the methods—we therefore discard the time-consuming optimization over the kernel subsets.

From the above analysis, the question arises why restricting the kernel set to the 15 BoW-S kernels affects the performance of the compared methods differently, for the VOC2009 and ImageCLEF2010 data sets. This can be explained by comparing the KKA/KTA scores of the kernels attained on VOC and on ImageCLEF (see Figure 1 (Right)): for the ImageCLEF data set the KTA scores are substantially more spread along all kernels; there is neither a dominance of the BoW-S subset in the KTA scores nor a particularly strong correlation within the BoW-S subset in the KKA scores. We attribute this to the less object-based and more ambiguous nature of many of the concepts contained in the ImageCLEF data set. Furthermore, the KKA scores for the ImageCLEF data (see Figure 1 (Left)) show that this dataset exhibits a higher variance among kernels—this is because the correlations between all kinds of kernels are weaker for the ImageCLEF data.

Therefore, because of this non-uniformity in the spread of the information content among the kernels, we can conclude that indeed our experimental setting falls into the situation where non-sparse MKL can outperform the baseline procedures (For example, the BoW features are more informative than HoG and HoC, and thus the uniform-sum-kernel-SVM is suboptimal. On the other hand, because of the fact that typical image features are only moderately informative, HoG and HoC still convey a certain amount of complementary information—this is what allows the performance gains reported in Tables 2 and 3.

Note that we class-wise normalized the KTA scores to sum to one. This is because we are rather interested in a comparison of the relative contributions of the particular kernels than in their absolute information content, which anyway can be more precisely derived from the AP scores already reported in Tables 2 and 3. Furthermore, note that we consider *centered* KKA and KTA scores, since it was argued in [53] that only those correctly reflect the test errors attained by established learners such as SVMs.

**Figure 2:**
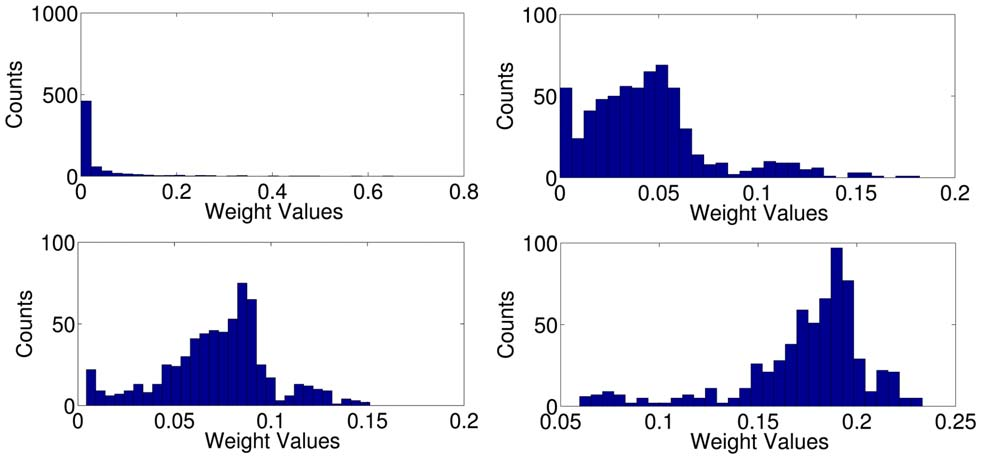
Histogram of kernel weights as output by 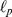
 **-norm MKL for the various classes on the VOC2009 data set (32 kernels×20 classes, resulting in 640 values).** 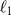-norm (top left)), 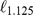-norm (top right), 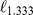-norm (bottom left), and 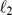-norm (bottom right).

**The Role of the Choice of** 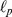 **-norm.** Next, we turn to the interpretation of the norm parameter 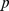 in our algorithm. We observe a big gap in performance between 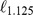-norm MKL and the sparse 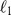-norm MKL. The reason is that for 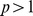 MKL is reluctant to set kernel weights to zero, as can be seen from Figure 2. In contrast, 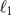-norm MKL eliminates 62.5% of the kernels from the working set. The difference between the 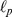-norms for 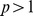 lies solely in the ratio by which the less informative kernels are down-weighted—they are never assigned with true zeros.

However, as proved in [23], in the computational optimum, the kernel weights are accessed by the MKL algorithm via the information content of the particular kernels given by a 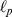-norm-dependent formula (see Eq. (12); this will be discussed in detail in Section *One Argument For the Sum Kernel: Randomness in Feature Extraction*). We mention at this point that the kernel weights all converge to the same, uniform value for 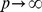. We can confirm these theoretical findings empirically: the histograms of the kernel weights shown in Figure 2 clearly indicate an increasing uniformity in the distribution of kernel weights when letting 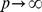. Higher values of 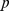 thus cause the weight distribution to shift away from zero and become slanted to the right while smaller ones tend to increase its skewness to the left.

Selection of the 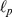-norm permits to tune the strength of the regularization of the learning of kernel weights. In this sense the sum-kernel SVM clearly is an extreme, namely fixing the kernel weights, obtained when letting 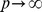. The sparse MKL marks another extreme case: 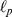-norms with 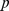 below 1 loose the convexity property so that 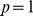 is the maximally sparse choice preserving convexity at the same time. Sparsity can be interpreted here that only a few kernels are selected which are considered most informative according to the optimization objective. Thus, the 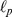-norm acts as a prior parameter for how much we trust in the informativeness of a kernel. In conclusion, this interpretation justifies the usage of 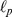-norm outside the existing choices 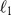 and 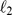. The fact that the sum-kernel SVM is a reasonable choice in the context of image annotation will be discussed further in Section *One Argument For the Sum Kernel: Randomness in Feature Extraction*.

Our empirical findings on ImageCLEF and VOC seem to contradict previous ones about the usefulness of MKL reported in the literature, where 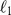 is frequently to be outperformed by a simple sum-kernel SVM (for example, see [32])—however, in these studies the sum-kernel SVM is compared to 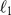-norm or 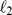-norm MKL only. In fact, our results *confirm* these findings: 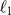-norm MKL is outperformed by the sum-kernel SVM in all of our experiments. Nevertheless, in this paper, we show that by using the more general 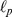-norm regularization, the prediction accuracy of MKL can be considerably leveraged, even clearly outperforming the sum-kernel SVM, which has been shown to be a tough competitor in the past [14]. But of course also the simpler sum-kernel SVM also has its advantage, although on the computational side only: in our experiments it was about a factor of ten faster than its MKL competitors. Further information about run times of MKL algorithms compared to sum kernel SVMs can be taken from [23].

**Figure 3:**
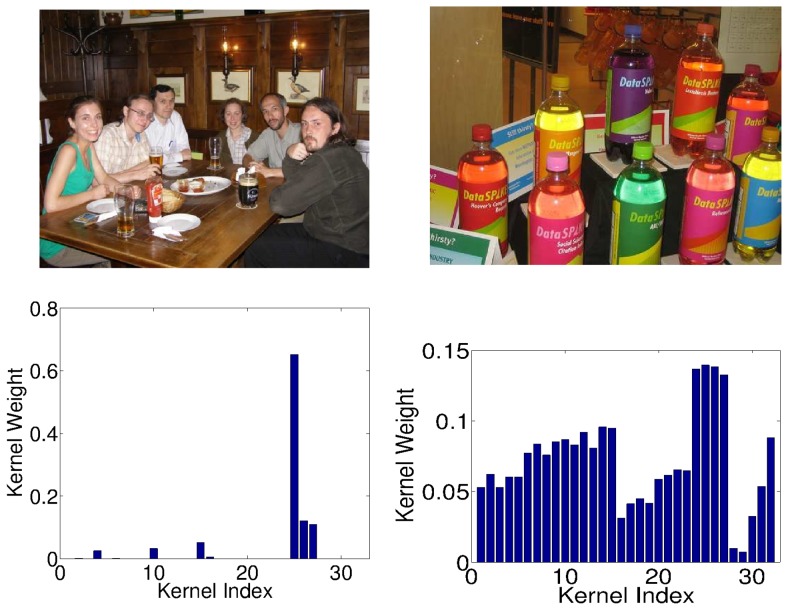
Images of typical highly ranked bottle images and kernel weights from 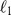
 **-MKL (left) and** 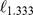 **-MKL (right).**

**Remarks for Particular Concepts.** Finally, we show images from classes where MKL helps performance and discuss relationships to kernel weights. We have seen above that the sparsity-inducing 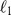-norm MKL clearly outperforms all other methods on the *bottle* class (see Table 1). Figure 3 shows two typical highly ranked images and the corresponding kernel weights as output by 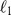-norm (Left) and 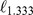-norm MKL (Right), respectively, on the bottle class. We observe that 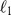-norm MKL tends to rank highly party and people group scenes. We conjecture that this has two reasons: first, many people group and party scenes come along with co-occurring bottles. Second, people group scenes have similar gradient distributions to images of large upright standing bottles sharing many dominant vertical lines and a thinner head section—see the left- and right-hand images in Figure 3. Sparse 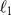-norm MKL strongly focuses on the dominant HoG product kernel, which is able to capture the aforementioned special gradient distributions, giving small weights to two HoC product kernels and almost completely discarding all other kernels.

**Figure 4:**
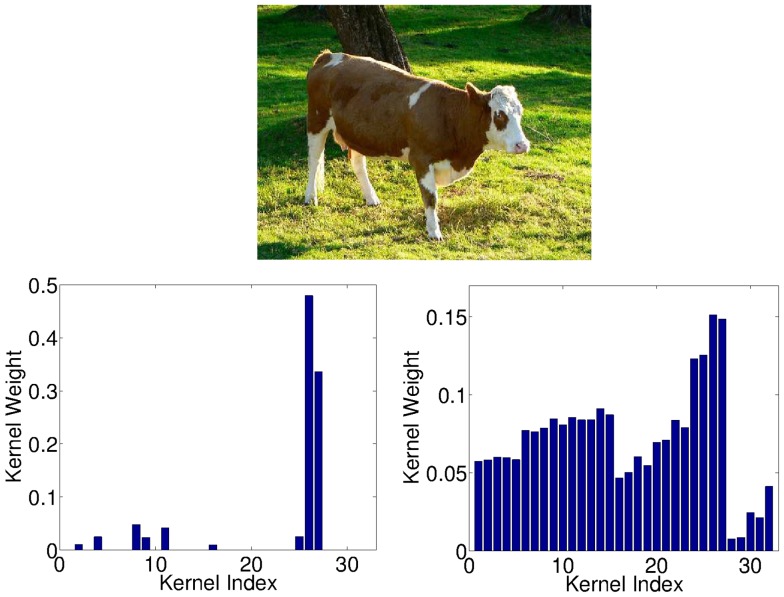
Images of a typical highly ranked cow image and kernel weights from 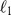
 **-MKL (left) and** 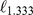 **-MKL (right).**

Next, we turn to the *cow* class, for which we have seen above that 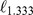-norm MKL outperforms all other methods clearly. Figure 4 shows a typical high-ranked image of that class and also the corresponding kernel weights as output by 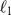-norm (Left) and 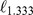-norm (Right) MKL, respectively. We observe that 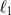-MKL focuses on the two HoC product kernels; this is justified by typical cow images having green grass in the background. This allows the HoC kernels to easily to distinguish the cow images from the indoor and vehicle classes such as *car* or *sofa*. However, horse and sheep images have such a green background, too. They differ in sheep usually being black-white, and horses having a brown-black color bias (in VOC data); cows have rather variable colors. Here, we observe that the rather complex yet somewhat color-based BoW-C and BoW-S features help performance—it is also those kernels that are selected by the non-sparse 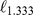-MKL, which is the best performing model on those classes. In contrast, the sum-kernel SVM suffers from including the five gray-channel-based features, which are hardly useful for the horse and sheep classes and mostly introduce additional noise. MKL (all variants) succeed in identifying those kernels and assign those kernels with low weights.

## Discussion

In the previous section we presented empirical evidence that 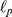-norm MKL considerably can help performance in visual image categorization tasks. We also observed that the gain is class-specific and limited for some classes when compared to the sum-kernel SVM, see again Tables 2 and 1. The same also holds for the ImageCLEF2010 dataset. In this section, we aim to shed light on the reasons of this behavior, in particular discussing strengths of the average kernel in Section *One Argument For the Sum Kernel: Randomness in Feature Extraction*, trade-off effects in Section *MKL and Prior Knowledge* and strengths of MKL in Section *One Argument for Learning the Multiple Kernel Weights: Varying Informative Subsets of Data*. Since these scenarios are based on statistical properties of kernels which can be observed in concept recognition tasks within computer vision we expect the results to be transferable to other algorithms which learn linear models over kernels such as [30], [31].

### One Argument For the Sum Kernel: Randomness in Feature Extraction

We would like to draw attention to one aspect present in BoW features, namely the amount of randomness induced by the visual word generation stage acting as noise with respect to kernel selection procedures.

**Experimental setup.** We consider the following experiment, similar to the one undertaken in [32]: we compute a BoW kernel ten times each time using the same local features, identical spatial pyramid tilings, and identical kernel functions; the only difference between subsequent repetitions of the experiment lies in the randomness involved in the generation of the codebook of visual words. Note that we use SIFT features over the gray channel that are densely sampled over a grid of step size six, 512 visual words (for computational feasibility of the clustering), and a 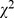 kernel. This procedure results in ten kernels that only differ in the randomness stemming from the codebook generation. We then compare the performance of the sum-kernel SVM built from the ten kernels to the one of the best single-kernel SVM determined by cross-validation-based model selection.

In contrast to [32] we try *two* codebook generation procedures, which differ by their intrinsic amount of randomness: first, we deploy 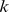-means clustering, with random initialization of the centers and a bootstrap-like selection of the best initialization (similar to the option ‘cluster’ in MATLAB's 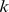-means routine). Second, we deploy *extremely randomized clustering forests* (ERCF) [55], [56], that are, ensembles of randomized trees—the latter procedure involves a considerably higher amount of randomization compared to 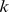-means.

**Results** **.** The results are shown in Table 6. For both clustering procedures, we observe that the sum-kernel SVM outperforms the best single-kernel SVM. In particular, this confirms earlier findings of [32] carried out for 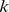-means-based clustering. We also observe that the difference between the sum-kernel SVM and the best single-kernel SVM is much more pronounced for ERCF-based kernels—we conclude that this stems from a higher amount of randomness is involved in the ERCF clustering method when compared to conventional 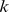-means. The standard deviations of the kernels in Table 6 confirm this conclusion. For each class we computed the conditional standard deviation
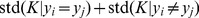
(10)averaged over all classes. The usage of a conditional variance estimator is justified because the ideal similarity in kernel target alignment (cf. equation (9)) does have a variance over the kernel as a whole however the conditional deviations in equation (10) would be zero for the ideal kernel. Similarly, the fundamental MKL optimization formula (12) relies on a statistic based on the two conditional kernels used in formula (10). Finally, ERCF clustering uses label information. Therefore averaging the class-wise conditional standard deviations over all classes is not expected to be identical to the standard deviation of the whole kernel.

**Table 6:** AP Scores and standard deviations showing amount of randomness in feature extraction: Results from repeated computations of BoW Kernels with randomly initialized codebooks.

Method	Best Single Kernel	Sum Kernel
VOC-KM	AP: 44.42±12.82	**45.84**±12.94
VOC-KM	Std: **30.81**	30.74
VOC-ERCF	AP: 42.60±12.50	**47.49**±12.89
VOC-ERCF	Std: **38.12**	37.89
CLEF-KM	AP: 31.09±5.56	**31.73**±5.57
CLEF-KM	Std: **30.51**	30.50
CLEF-ERCF	AP: 29.91±5.39	**32.77**±5.93
CLEF-ERCF	Std: **38.58**	38.10

VOC-KM denotes VOC2009 dataset and k-means for visual word generation, VOC-ERCF denotes VOC2009 dataset and ERCF for visual word generation. Similarly CLEF denotes ImageCLEF2010 dataset.

We observe in Table 6 that the standard deviations are lower for the sum kernels. Comparing ERCF and k-means shows that the former not only exhibits larger absolute standard deviations but also greater differences between single-best and sum-kernel as well as larger differences in AP scores.

We can thus postulate that the reason for the superior performance of the sum-kernel SVM stems from averaging out the randomness contained in the BoW kernels (stemming from the visual-word generation). This can be explained by the fact that averaging is a way of reducing the variance in the predictors/models [57]. We can also remark that such variance reduction effects can also be observed when averaging BoW kernels with varying color combinations or other parameters; this stems from the randomness induced by the visual word generation.

Note that in the above experimental setup each kernel uses the *same* information provided via the local features. Consequently, the best we can do is *averaging*—learning kernel weights in such a scenario is likely to suffer from overfitting to the noise contained in the kernels and can only decrease performance.

To further analyze this, we recall that, in the computational optimum, the information content of a kernel is measured by 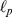-norm MKL via the following quantity, as proved in [23]:
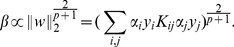
(11)In this paper we deliver a novel interpretation of the above quantity; to this end, we decompose the right-hand term into two terms as follows:
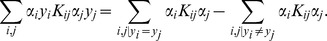
The above term can be interpreted as a difference of the support-vector-weighted sub-kernel restricted to consistent labels *and* the support-vector-weighted sub-kernel over the opposing labels. Equation (11) thus can be rewritten as
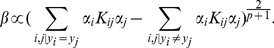
(12)Thus, we observe that random influences in the features combined with overfitting support vectors can suggest a falsely high information content in this measure for *some* kernels. SVMs do overfit on BoW features. Using the scores attained on the training data subset we can observe that many classes are deceptive-perfectly predicted with AP scores fairly above 0.9. At this point, non-sparse 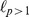-norm MKL offers a parameter 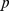 for regularizing the kernel weights—thus hardening the algorithm to become robust against random noise, yet permitting to use some degree of information given by Equation (12).

[32] reported in accordance to our idea about overfitting of SVMs that 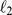-MKL and 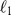-MKL show no gain in such a scenario while 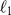-MKL even reduces performance for some datasets. This result is not surprising as the overly sparse 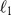-MKL has a stronger tendency to overfit to the randomness contained in the kernels/feature generation. The observed amount of randomness in the state-of-the-art BoW features could be an explanation why the sum-kernel SVM has shown to be a quite hard-to-beat competitor for semantic concept classification and ranking problems.

### MKL and Prior Knowledge

For solving a learning problem, there is nothing more valuable than *prior knowledge*. Our empirical findings on the VOC2009 and ImageCLEF09 data sets suggested that our experimental setup was actually biased towards the sum-kernel SVM via usage of prior knowledge when choosing the set of kernels/image features. We deployed kernels based on four features types: BoW-S, BoW-C, HoC and HoG. However, the *number* of kernels taken from each feature type is not equal. Based on our experience with the VOC and ImageCLEF challenges we used a higher fraction of BoW kernels and less kernels of other types such as histograms of colors or gradients because we already knew that BoW kernels have superior performance.

To investigate to what extend our choice of kernels introduces a bias towards the sum-kernel SVM, we also performed another experiment, where we deployed a higher fraction of weaker kernels for VOC2009. The difference to our previous experiments lies in that we summarized the 15 BOW-S kernels in 5 product kernels reducing the number of kernels from 32 to 22. The results are given in Table 7; when compared to the results of the original 32-kernel experiment (shown in Table 2), we observe that the AP scores are in average about 4 points smaller. This can be attributed to the fraction of weak kernels being higher as in the original experiment; consequently, the gain from using (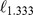-norm) MKL compared to the sum-kernel SVM is now more pronounced: over 2 AP points—again, this can be explained by the higher fraction of weak (i.e., noisy) kernels in the working set.

**Table 7:** MKL versus Prior Knowledge: AP Scores with a smaller fraction of well scoring kernels.

Class/ 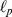 -norm	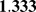	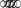
Aeroplane	**77.82**±7.701	76.28±8.168
Bicycle	**50.75**±11.06	46.39±12.37
Bird	**57.7**±8.451	55.09±8.224
Boat	**62.8**±13.29	60.9±14.01
Bottle	**26.14**±9.274	25.05±9.213
Bus	**68.15**±22.55	67.24±22.8
Car	**51.72**±8.822	49.51±9.447
Cat	**56.69**±9.103	55.55±9.317
Chair	**51.67**±12.24	49.85±12
Cow	**25.33**±13.8	22.22±12.41
Diningtable	**45.91**±19.63	42.96±20.17
Dog	**41.22**±10.14	39.04±9.565
Horse	**52.45**±13.41	50.01±13.88
Motorbike	**54.37**±12.91	52.63±12.66
Person	**80.12**±10.13	79.17±10.51
Pottedplant	**35.69**±13.37	34.6±14.09
Sheep	**37.05**±18.04	34.65±18.68
Sofa	**41.15**±11.21	37.88±11.11
Train	**70.03**±15.67	67.87±16.37
Tvmonitor	**59.88**±10.66	57.77±10.91
Average	**52.33**±12.57	50.23±12.79

In summary, this experiment should remind us that semantic classification setups use a substantial amount of prior knowledge. Prior knowledge implies a *pre-selection* of highly effective kernels—a carefully chosen set of strong kernels constitutes a bias towards the sum kernel. Clearly, pre-selection of strong kernels reduces the need for learning kernel weights; however, in settings where prior knowledge is sparse, statistical (or even adaptive, adversarial) noise is inherently contained in the feature extraction—thus, beneficial effects of MKL are expected to be more pronounced in such a scenario.

### One Argument for Learning the Multiple Kernel Weights: Varying Informative Subsets of Data

In the previous sections, we have presented evidence for why the sum-kernel SVM is considered to be an efficient learner in visual image categorization. Nevertheless, in our experiments we have observed gains in accuracy by using MKL for many concepts. In this section, we investigate causes for this performance gain.

We formulate a hypothesis for the performance gains achieved by MKL: each kernel is informative for a subset of the data in the sense that the kernel classifies that subset well. These subsets can be partially disjoint between kernels and have varying sizes. The MKL information criterion given in Eq. (12) is able to exploit such differences in informative subsets and is able to weight kernels properly despite being a *global* information measure that is computed over the support vectors (which in turn are chosen over the *whole dataset*).

In this section, we will present evidence for this hypothesis in two steps. In the first step we show that our kernels computed from the real ImageCLEF2010 dataset indeed have fairly disjoint informative subsets. This suggests that our observed performance gains achieved by MKL could be explained by MKL being able to exploit such a scenario. In the second step we will create a toy dataset such that the informative subsets of kernels are disjoint by design. We will show that, in this controlled toy scenario, MKL outperforms average-kernel SVMs in a statistically significant manner. These two steps together will serve as evidence for our hypothesis given above.

The main question for the first step is how to determine which set of samples is informative for a given kernel matrix and how to measure the diversity of two sets defined by two kernels. Despite using ranking measures for most of the paper, we will stick here to a simple definition. Consider one binary classification problem. The set of all true positively and true negatively classified test examples using a SVM will be the informative subset for a kernel. If we restrict the kernel to the union of these two subsets of the test data set, then the resulting classifier would discriminate the two classes perfectly. Since we do not have test data labels for the Pascal VOC dataset, we will restrict ourselves to the ImageCLEF data.

The diversity measure will be defined in two steps: at first for two sets, then for a pair of kernels. The diversity measure 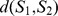 for two sets 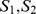 should have two properties: it should be 1 if these sets are maximally disjoint and be equal to zero if one set is contained in the other. The second property follows the idea that if the informative set of one kernel is contained in the informative set of another, then the first kernel is inferior to the second and we would like to reflect this in our diversity measure by setting it to zero as we would expect little gain from adding the first kernel to the second one in SVMs or MKL algorithms – we would say the inferior kernel does not add any diversity.

Using these two conditions we note that two sets 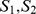 are maximally disjoint if 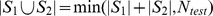,where 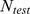 is the total number of test samples. Analogously, if one set is contained in the other, then 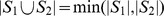. Linear interpolation between these two extremes yield the diversity measure for a pair of sets 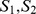:
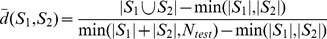
(13)Note that we do not use the symmetric difference here because this would be non-empty if one set was contained in the other.

The diversity measure 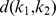 for two kernels 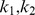, still given a fixed binary classification problem, will be defined as the sum of the diversities between the two true positive sets from both kernels and the two true negative sets from both kernels. Let 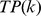 be the set of true positive samples of kernel 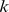, and 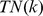 the corresponding set of true negative samples. Then we define
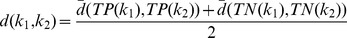
(14)Treating true positives and true negatives separately makes sense because for most of the classes the positive labeled samples constitute only a small fraction of all samples which has its impact on the maximal number of true positives.

**Figure 5:**
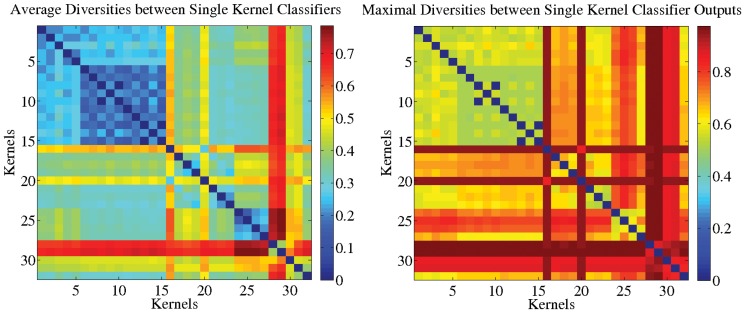
Diversity measure between correctly classified samples for all pairs of 32 kernels. Left: Average over all concept classes. Right: Maximum over all concept classes.

Since the ImageCLEF2010 dataset has 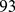 classes, we consider the average diversity of a pair of kernels over all classes and the maximal diversity over all classes. Figure 5 shows both diversities. We can see an interesting phenomenon: the diversities are low between the first 15 BoW-S kernels. This may serve as an explanation for anecdotal experiences that using MKL on BoW-S features alone yields no gains. The diversity is low but the randomness in feature extraction as discussed in a subsection above results in overfitting. However for the whole kernel set of all 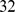 kernels the diversities are large. The mean average diversity (when the mean is computed over all pairs of kernels and the average of all 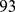 binary classification problems) is 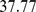, the mean maximal diversity over all kernel pairs is 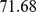 when the maximum is computed over all 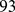 binary classification problems. This concludes the first step: our kernel set does have partially disjoint sets of true positive and true negative samples between pairs of kernels. The informative subsets of kernels are fairly disjoint.

In the second step we will construct two toy data sets in which by design we have kernels with disjoint informative subsets of varying sizes. The goal is to show that MKL outperforms the average kernel SVM under such conditions. This implies that the MKL information criterion given in Eq. (12) is able to capture such differences in informative subsets despite being a *global* information measure. In other words, the kernel weights are global weights that uniformly hold in all regions of the input space. While on the first look it appears to be a disadvantage, explicitly finding informative subsets of the input space on real data may not only imply a too high computational burden (note that the number of partitions of an 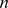-element training set is exponential in 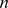) but also is very likely to lead to overfitting.

We performed the following toy experiment. The coarse idea is that we create 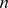 features of dimension 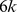, where 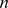 is the number of data samples. We will compute 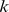 kernels such that the i-th kernel is computed only from the i-th consecutive block of 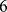 feature dimensions from all available 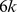 dimensions. We want the i-th kernel to have an informative subset of samples and an uninformative complement. After drawing labels for all 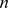 samples, we partition all data samples into 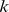 blocks of varying size. The precise sizes of the blocks 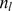 will be given below. The i-th block of data samples will be the informative subset for the i-th kernel. This will be achieved in the following way: for the i-th block of samples the i-th block of dimensions will be drawn from two Gaussians having different means such that the chosen Gaussian depends on the label of the data sample. This implies that each of the two Gaussians is responsible for creating the samples of one label. For all other samples (except for the i-th block of samples) the i-th block of dimensions will be drawn from an unconditional mixture of two Gaussians, i.e. which Gaussian is used will be independent of the sample labels. Therefore the i-th kernel which is computed from the i-th block of dimensions contains discriminative information only for the samples coming from the i-th block of samples. For all other samples, the i-th kernel uses features from a mixture of Gaussians independent of the sample labels which allows no discrimination of labels. By this construction the i-th kernel will have the i-th set of samples as discriminative subset. Furthermore, all kernels will have mutually disjoint informative subsets, because the i-th kernel is discriminative only on the i-th subset.

We generated a fraction of 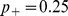 of positively labeled and 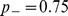 of negatively labeled training examples (motivated by the unbalancedness of training sets usually encountered in computer vision). The precise data creation protocol is given in the experimental section parts for experiments one and two.

We consider two experimental setups for sampling the data, which differ in the number of employed kernels 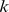 and the sizes of the informative sets. In both cases, the informative features are drawn from two sufficiently distant normal distributions (one for each class) while the uninformative features are just Gaussian noise (mixture of Gaussians). The experimental setup of the first experiment can be summarized as follows:

**Experimental Settings for Experiment 1 (k = 3 kernels).** Let 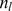 be the size of the l-th informative subset and 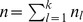 the total sample size. 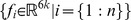 are the features to be drawn where 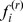 is the r-th dimension of the i-th feature.
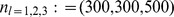


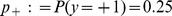


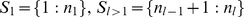


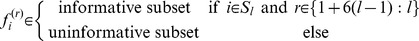
(15)The features for the informative subset are drawn according to
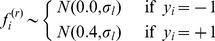
(16)

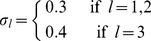
(17)The features for the uninformative subset are drawn according to
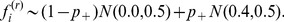
(18)Finally the l-th kernel is defined as
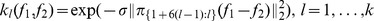
(19)where 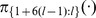 is the projection on the feature dimensions ranging in the set 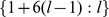.

For Experiment 1 the three kernels had disjoint informative subsets of sizes 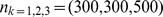. We used 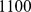 data points for training and the same amount for testing. We repeated this experiment 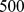 times with different random draws of the data.

Note that the features used for the uninformative subsets are drawn as a mixture of the Gaussians with a higher variance, though. The increased variance encodes the assumption that the feature extraction produces unreliable results on the uninformative data subset. None of these kernels are pure noise or irrelevant. Each kernel is the only informative one for its own informative subset of data points.

We now turn to the experimental setup of the second experiment which is an extension to five kernels:

**Experimental Settings for Experiment 2 (k = 5 kernels).** Let 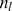 be the size of the l-th informative subset and 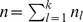 the total sample size. 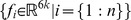 are the features to be drawn where 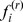 is the r-th dimension of the i-th feature.
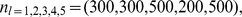


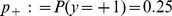


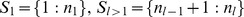


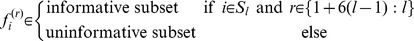
(20)The features for the informative subset are drawn according to
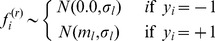
(21)

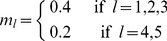
(22)

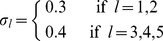
(23)The features for the uninformative subset are drawn according to
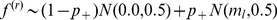
(24)Finally the l-th kernel is defined as
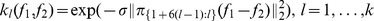
(25)where 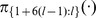 is the projection on the feature dimensions ranging in the set 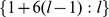.

As for the real experiments, we normalized the kernels to having standard deviation 1 in Hilbert space and optimized the regularization constant by grid search in 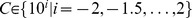.

Table 8 shows the results. The null hypothesis of equal means is rejected by a t-test with a p-value of 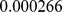 and 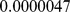, respectively, for Experiment 1 and 2, which is highly significant.

**Table 8:** AP Scores in Toy experiment using Kernels with disjoint informative subsets of Data.

Setup	ℓ_∞_  -SVM	**ℓ_1.0625_**  -MKL	t-test p-value
1	68.72±3.27	69.49±3.17	0.000266
2	55.07±2.86	56.39±2.84	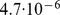

Experiment 2 shows that the design of the Experiment 1 is no singular lucky find: we can extend the setting of experiment 1 and observe similar results again when using more kernels; the performance gaps then even increased. Experiment 2 uses five kernels instead of just three. Again, the informative subsets are disjoint, but this time of sizes 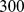, 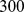, 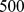, 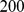, and 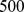; the the Gaussians are centered at 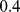, 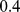, 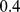, 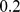, and 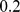, respectively, for the positive class; and the variance is taken as 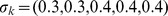. Compared to Experiment 1, this results in even bigger performance gaps between the sum-kernel SVM and the non-sparse 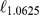-MKL. One can imagine to create learning scenarios with more and more kernels in the above way, thus increasing the performance gaps—since we aim at a relative comparison, this, however, would not further contribute to validating or rejecting our hypothesis.

Furthermore, we also investigated the single-kernel performance of each kernel: we observed the best single-kernel SVM (which attained AP scores of 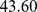, 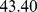, and 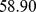 for Experiment 1) being inferior to both MKL (regardless of the employed norm parameter 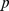) and the sum-kernel SVM over the whole set of kernels. The differences were significant with fairly small p-values (for example, for 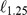-MKL the p-value was still about 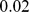).

We emphasize that we did not design the example in order to achieve a maximal performance gap between the non sparse MKL and its competitors. For such an example, see the toy experiment of [23].Our focus here was to confirm our hypothesis that kernels in semantic concept classification are based on varying informative subsets of the data—although MKL computes global weights, it emphasizes on kernels that are relevant on the largest informative set and thus approximates the infeasible combinatorial problem of computing an optimal partition/grid of the space into regions which underlie identical optimal weights. Though, in practice, we expect the situation to be more complicated as informative subsets may overlap between kernels instead of being disjoint as modeled here

Nevertheless, our hypothesis also opens the way to new directions for learning of kernel weights, namely restricted to subsets of data chosen according to a meaningful principle. Finding such principles is one the future goals of MKL—we sketched one possibility: locality in feature space. A first starting point may be the work of [58], [59] on localized MKL.

We conclude the second step. MKL did outperform the average kernel SVM in this controlled toy data scenario with disjoint informative subsets for each kernel. It may serve as empirical evidence for our hypothesis why we observe gains using MKL on real data: MKL with its global information criterion can exploit scenarios in which each kernel is informative for a subset of the data and these subsets are partially disjoint between kernels.

## Conclusions

Analyzing images using many different features is a common strategy in visual object recognition. This raises the question of *how* to combine these features. In this paper, we revisited this important topic and discussed machine learning approaches to adaptively combine different image features in a systematic and theoretically well founded manner. While MKL approaches in principle solve this problem it has been observed that the standard 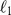-norm based MKL often cannot outperform SVMs that use an average of a large number of kernels. One hypothesis why this seemingly unintuitive result may occur is that the sparsity prior may not be appropriate in many real world problems—especially, when prior knowledge is already at hand. We tested whether this hypothesis holds true for computer vision and applied the recently developed non-sparse 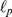 MKL algorithms to object classification tasks. The 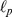-norm constitutes a slightly less severe method of sparsification. By choosing 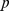 as a hyperparameter, which controls the degree of non-sparsity and regularization, from a set of candidate values with the help of a validation data, we showed that 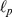-MKL significantly improves SVMs with averaged kernels and the standard sparse 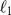 MKL.

Future work will study localized MKL and methods to include hierarchically structured information into MKL, e.g. knowledge from taxonomies for multi-label ranking [60], [61] or the classical multi-class classification, semantic information or spatial priors. Another interesting direction is MKL-KDA [29], [30]. The difference to the method studied in the present paper lies in the base optimization criterion: KDA [62] leads to non-sparse solutions in 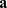 while ours leads to sparse ones (i.e., a low number of support vectors). While on the computational side the latter is expected to be advantageous, the first one might lead to more accurate solutions. We expect the regularization over kernel weights (i.e., the choice of the norm parameter 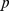) having similar effects for MKL-KDA like for MKL-SVM. Future studies will expand on that topic. First experiments on ImageCLEF2010 show for sum kernel SRKDA [63] a result of 39.29 AP points which is slightly better than the sum kernel results for the SVM (39.11 AP) but worse than MKL-SVM.

## Supporting Information

Table S1
**The file Table S1 contains AP scores on ImageCLEF2010 test data with fixed** 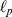 **-norm for each of the 93 concept classes listed separately.**
(PDF)
