# Supplementary material for: Insights from Classifying Visual Concepts with Multiple Kernel Learning
Source: PLoS One. 2012 Aug 24;7(8):e38897. doi: 10.1371/journal.pone.0038897 (PMC3427351; doi:10.1371/journal.pone.0038897)
Supplement: Table S1 — The file Table S1 contains AP scores on ImageCLEF2010 test data with fixed -norm for each of the 93 concept classes listed separately. (PDF) [file pone.0038897.s001.pdf]

Supplement to: Insights from Classifying Visual Concepts with Multiple Kernel Learning

Alexander Binder[1,2,*], Shinichi Nakajima[3], Marius Kloft[1], Christina Müller[1], Wojciech Samek[1,2], Ulf Brefeld[4,5], Klaus-Robert Müller[1,6,7], Motoaki Kawanabe[8]
1 Machine Learning Group, Berlin Institute of Technology, Berlin, Germany
2 Fraunhofer Institute FIRST, Berlin, Germany
3 Optical Research Laboratory, Nikon Corporation, Tokyo, Japan
4 Knowledge Discovery and Machine Learning Group, University of Bonn, Bonn, Germany
5 Zalando GmbH, Berlin, Germany
6 Bernstein Focus: Neurotechnology Berlin, Berlin, Germany
7 Department of Brain and Cognitive Engineering, Korea University, Anam-dong, Seongbuk-gu, Seoul 136-713, Seoul, Korea
8 ATR Research, Kyoto, Japan
∗ E-mail: alexander.binder@tu-berlin.de

Abstract

This supplement delivers the average precision scores for the ImageCLEF2010 test dataset listed for all 93 visual concepts and all ℓ_p-norms used including the average kernel as the special case ℓ^∞.

Tables

Table 1. AP scores on ImageCLEF2010 test data with fixed ℓ_p-norm. Part 1.

	Partylife	FamilyFriends	Beach	BuildSights	Snow	Citylife
ℓ^1	28.41	50.82	39.36	54.94	12.75	50.14
$\ell^{1.125}$	30.52	**52.55**	**42.75**	**57.23**	19.97	52.79
$\ell^{1.333}$	**30.84**	52.26	42.71	56.87	20.38	**52.8**
ℓ^2	30.46	51.54	41.77	55.72	19.94	52.34
ℓ^∞	30.55	50.76	40.78	55.26	**20.49**	51.69
	**Landscape**	**Sports**	**Desert**	**Spring**	**Summer**	**Autumn**
ℓ^1	81.42	7.464	10.85	5.962	28.39	26.12
$\ell^{1.125}$	**81.97**	**10.37**	15.3	13.52	29.12	32.79
$\ell^{1.333}$	81.8	10.33	15.12	15.59	**29.42**	33.49
ℓ^2	81.48	10.19	**16.55**	16	29.34	33.26
ℓ^∞	81.16	10.07	15.82	**16.54**	29.3	**33.58**
	**Winter**	**NoSeason**	**Indoor**	**Outdoor**	**NoPlace**	**Plants**
ℓ^1	15.66	96.51	61.8	90.79	60.1	78.04
$\ell^{1.125}$	19.49	96.61	**62.53**	91.39	60.65	79.28
$\ell^{1.333}$	**20.11**	**96.61**	62.44	**91.49**	**60.92**	**79.44**
ℓ^2	20.09	96.53	62.12	91.43	60.33	79.23
ℓ^∞	19.81	96.47	61.69	91.26	60.06	78.85
	**Flowers**	**Trees**	**Sky**	**Clouds**	**Water**	**Lake**
ℓ^1	43.25	63.03	91.39	87.65	62.69	26.24
$\ell^{1.125}$	46.42	65.35	**91.8**	**88**	65.43	26.95
$\ell^{1.333}$	47.47	**65.39**	91.73	87.93	**66.03**	**27.13**
ℓ^2	47.89	64.87	91.64	87.77	66.01	26.92
ℓ^∞	**47.91**	64.13	91.39	87.54	65.79	25.79
	**River**	**Sea**	**Mountains**	**Day**	**Night**	**NoTime**
ℓ^1	15.68	47.55	**53.21**	88.03	55.89	80.1
$\ell^{1.125}$	**19.75**	48.74	52.86	88.68	57.85	80.83
$\ell^{1.333}$	18.92	**48.79**	51.95	**88.69**	**58.19**	**80.83**
ℓ^2	18.57	48.19	51.03	88.54	58.13	80.62
ℓ^∞	17.8	47.77	50.36	88.4	57.85	80.38
	**Sunny**	**Sunset**	**StillLife**	**Macro**	**Portrait**	**Overexpos**
ℓ^1	46.51	81.16	37.64	48.5	65.58	17.43
$\ell^{1.125}$	49.82	**81.58**	**40.72**	**50.2**	67.58	**19.9**
$\ell^{1.333}$	**50.13**	81.37	40.65	49.66	**67.62**	18.9
ℓ^2	49.97	81.09	39.76	49.07	67.24	18.51
ℓ^∞	50.08	80.77	39.54	50.02	66.72	17.61
	**Underexpos**	**NeutralIllum**	**MotionBlur**	**Outoffocus**	**PartBlur**	**NoBlur**
ℓ^1	27.74	98.38	13.35	10.28	72.37	90.92
$\ell^{1.125}$	28	98.4	**19.82**	**15.08**	**74.26**	**91.39**
$\ell^{1.333}$	27.43	98.31	19.72	14.88	74.2	91.14
ℓ^2	26.99	98.26	19.22	14.21	73.8	91.21
ℓ^∞	**29.22**	**98.49**	18.47	13.47	73.31	91.06
	**SinglePers**	**SmallGroup**	**BigGroup**	**NoPersons**	**Animals**	**Food**
ℓ^1	54.52	30.74	34.31	91.5	44.24	49.57
$\ell^{1.125}$	**55.85**	**32.88**	41.11	91.99	49.78	52.73
$\ell^{1.333}$	55.78	32.78	**41.81**	**92.03**	**50.08**	**53.31**
ℓ^2	55.34	32.28	41.29	92	49.78	53.26
ℓ^∞	54.81	31.83	40.5	91.81	49.17	52.81

Table 2. AP scores on ImageCLEF2010 test data with fixed ℓ_p-norm. Part 2.

	Vehicle	Aesthetic	OverallQuality	Fancy	Architecture	Street
ℓ^1	45.17	**28.63**	**22.6**	17.14	27.04	29.46
$\ell^{1.125}$	**47.62**	28.25	22.41	17.95	28.8	33.7
$\ell^{1.333}$	47.35	27.14	21.57	17.15	**29.25**	**33.91**
ℓ^2	47	26.01	20.77	16.92	28.91	33.42
ℓ^∞	46.25	28.34	22.46	**18.82**	27.84	32.79
	Church	Bridge	ParkGarden	Rain	Toy	MusicInstr
ℓ^1	5.29	5.087	42.02	0.6378	15.27	5.066
$\ell^{1.125}$	**8.15**	7.437	44.44	0.8926	22.05	5.231
$\ell^{1.333}$	7.441	**7.546**	**44.75**	0.9725	**22.35**	5.445
ℓ^2	6.577	7.243	44.53	0.9875	21.97	**5.609**
ℓ^∞	6.241	7.117	43.91	**1.017**	20.58	5.33
	Shadow	Bodypart	Travel	Work	Birthday	VisualArt
ℓ^1	**11.23**	22.46	11.68	4.264	**1.143**	32.98
$\ell^{1.125}$	10.93	23.84	**12.89**	**4.596**	0.9434	32.99
$\ell^{1.333}$	10.15	**24.15**	12.49	4.468	0.9152	32.62
ℓ^2	9.702	23.63	12.33	4.314	0.8556	31.97
ℓ^∞	10.89	23.07	12.69	4.257	0.8731	**33.05**
	Graffiti	Painting	Artificial	Natural	Technical	Abstract
ℓ^1	3.411	12.66	12.64	71.16	5.979	**2.553**
$\ell^{1.125}$	**4.467**	18.57	13.96	**71.66**	**6.107**	2.33
$\ell^{1.333}$	4.273	18.83	13.67	71.64	5.853	2.137
ℓ^2	4.094	18.9	13.18	70.62	5.82	2.099
ℓ^∞	3.882	**19.58**	**13.97**	71.32	6.01	2.025
	Boring	Cute	Dog	Cat	Bird	Horse
ℓ^1	7.281	**59.58**	22.04	2.132	13.02	1.48
$\ell^{1.125}$	**7.68**	59.13	31.54	8.586	23.87	**4.414**
$\ell^{1.333}$	7.388	59.46	**31.99**	**8.97**	**23.98**	3.931
ℓ^2	7.23	58.08	31.85	8.208	23.33	3.408
ℓ^∞	7.167	58.88	31.11	7.626	22.7	3.279
	Fish	Insect	Car	Bicycle	Ship	Train
ℓ^1	0.915	11.51	31.27	18.9	8.157	12.97
$\ell^{1.125}$	**1.844**	**16.2**	**34**	**26.17**	**9.749**	**15.42**
$\ell^{1.333}$	1.684	15.6	33.89	26.13	9.164	14.4
ℓ^2	1.594	14.94	33.51	25.53	8.688	13.45
ℓ^∞	1.605	15.06	32.54	24.5	8.581	12.48
	Airplane	Skateboard	Female	Male	Baby	Child
ℓ^1	5.913	0.2205	44.4	20.65	8.028	6.304
$\ell^{1.125}$	11.08	**0.4211**	**45.78**	21.02	17.85	10.36
$\ell^{1.333}$	**11.14**	0.41	45.51	21.01	**18.14**	**11.01**
ℓ^2	10.22	0.3963	44.78	**21.03**	17.12	10.8
ℓ^∞	10.18	0.4172	43.58	20.86	15.22	10.67
	Teenager	Adult	Oldperson			
ℓ^1	21.32	53.03	5.068			
$\ell^{1.125}$	23.69	**54.33**	5.624			
$\ell^{1.333}$	23.35	53.96	**5.66**			
ℓ^2	23.03	53.4	5.58			
ℓ^∞	**23.78**	53	5.46			
